# Deubiquitinase Inhibitor b-AP15 Attenuated LPS-Induced Inflammation via Inhibiting ERK1/2, JNK, and NF-Kappa B

**DOI:** 10.3389/fmolb.2020.00049

**Published:** 2020-04-22

**Authors:** Fangcheng Zhang, Ruqin Xu, Renjie Chai, Qiong Xu, Mingke Liu, Xuke Chen, Xiaohua Chen, Tianyu Kong, Chongyu Zhang, Shi-Ming Liu, Zhenhui Zhang, Ningning Liu

**Affiliations:** ^1^Guangzhou Institute of Cardiovascular Disease, Guangdong Key Laboratory of Vascular Diseases, State Key Laboratory of Respiratory Disease, The Second Affiliated Hospital, Guangzhou Medical University, Guangzhou, China; ^2^Department of Critical Care Medicine, The Second Affiliated Hospital, Guangzhou Medical University, Guangzhou, China

**Keywords:** inflammation, deubiquitinase inhibitor, b-AP15, ERK1/2, JNK, NF-κB

## Abstract

b-AP15 is a deubiquitinase (DUB) inhibitor of 19S proteasomes, which in turn targets ubiquitin C-terminal hydrolase 5 (UCHL5) and ubiquitin-specific peptidase 14 (USP14). Nuclear factor kappa B (NF-κB) is closely linked to cellular response in macrophages when the organism is in the state of microbial infection, and it acts as a vital part in the mechanism of inflammatory reaction. However, the molecular mechanism by which DUB inhibitors, especially b-AP15, regulates inflammation remains poorly understood. This study aimed to investigate the relationship between b-AP15 and inflammation. The results showed that b-AP15 treatment significantly reduced the amounts of inflammatory indicators, such as tumor necrosis factor alpha (TNF-α) and interleukin 6 (IL-6) in lipopolysaccharide (LPS)-stimulated THP-1 and macrophages. Meanwhile, similar results were obtained from *in vivo* experiments. In addition, b-AP15 also significantly improved the survival rate of sepsis mouse via high-density LPS mediation. Furthermore, b-AP15 also inhibited the ERK1/2 and JNK phosphorylation, increased IκBα levels, and inhibited NF-κB p65 by removing them from the cytoplasm into the nucleus. All these findings suggested that b-AP15 has anti-inflammatory action and acts as a potential neoteric target drug for treating microbial infection.

## Introduction

Inflammation is generally one of the most common clinical symptoms that are triggered in response to damage caused to living tissues. It involves an array of complex reactions, including biological and biochemical reactions (Medzhitov, 2008; Leyva-Lopez et al., 2016). The molecular mechanisms associated with inflammatory response are complex. Among them, the nuclear factor kappa B (NF-κB) and mitogen-activated protein kinase (MAPK) signaling pathways play important roles in inflammatory response. Previous studies have reported the existence of NF-κB in various animal cells. It is a protein complex that controls gene transcription and regulates genetic expression of many inflammatory cytokines (Gilmore, 2006; Carmody and Chen, 2007; Hayden and Ghosh, 2012). IκBα is an inhibitor of NF-κB that is present in the cytoplasm. It normally binds to NF-κB and inactivates them in the cytoplasm. When organisms respond to harmful stimuli, the IκBα is phosphorylated by the IκB kinase (IKK) complex quickly. Based on the phosphorylation, the IκBα is attached with a polyubiquitin chain labeled by the SCF-β-TrCP E3 ligase, which is then transferred and degraded in the proteasome. Meanwhile, degradation of IκBα led to the exposure of nuclear localization signals (NLSs) of NF-κB. Upon NLS, the NF-κB dimers are transferred to the nucleus and turned on the specific gene expression of proinflammatory cytokines (Gilmore, 1999; Hayden and Ghosh, 2004; Brasier, 2006; Perkins, 2007; Solt and May, 2008). Besides, the MAPK pathway is considered vital for various cellular functions, such as cell growth, proliferation, and differentiation. The MAPK pathway encompasses the signaling molecules ERK1/2, JNK, and p38 MAPK. MAPK is induced by multiple stimulating factors, causing a downstream signaling cascade. It plays a critical role in the regulation of inflammation (Arthur and Ley, 2013; Paunovic and Harnett, 2013).

The ubiquitin–proteasome system (UPS) is the major pathway for protein degradation in eukaryotes. In short, this process targets the proteins to undergo ubiquitination by covalent conjugation of ubiquitin chains, and the proteasome then recognizes and degrades the ubiquitin-tagged proteins. However, the ubiquitin chains are cleaved by deubiquitinase (DUB) before undergoing degradation of ubiquitin-tagged proteins by the proteasome (Hershko and Ciechanover, 1992; Hochstrasser, 1995). The DUBs participate in multiple signal transduction pathways and biological functions, such as growth and differentiation, stress response, apoptosis, damage, and repair (Song and Rape, 2008; Hussain et al., 2009; Aressy et al., 2010). Among them, ubiquitin-specific peptidase 14 (USP14), Rpn11, and UCH37 are associated with mammalian 19S proteasomes and play a vital role (D’Arcy et al., 2011; Liu et al., 2019). At times, specific DUBs are highly expressed in some diseases, such as tumors and cardiovascular diseases. Perhaps this suggests that the DUBs can likely act as potential drug targets for clinical treatment (Wu et al., 2013; Liu et al., 2016; Liao et al., 2019; Xia et al., 2019).

b-AP15 is a DUB inhibitor that acts on 19S proteasomes of USP14 and does not impact the 20S proteasomes. Therefore, theoretically, it might have fewer side effects (Tian et al., 2014; Wang et al., 2015). b-AP15 leads to multiple cellular efficiency in several individual components of cells. According to some previous research studies, it could inhibit cancer cell proliferation and induce cancer cell apoptosis in several kinds of cancers (Cai et al., 2017; Ding et al., 2018; Kropp et al., 2018; Xia et al., 2018). In addition, in our previous research, we also confirmed that the DUB inhibitor IU1 successfully suppressed lipopolysaccharide (LPS)-induced inflammatory reaction (Liu et al., 2017). In this study, we proved that the DUB inhibitor b-AP15 attenuated LPS-induced inflammation via blocking the phosphorylation of ERK1/2, JNK, and NF-κB activation. This in turn provides a potential neoteric drug target for treating microbial infection.

## Materials and Methods

### Materials

b-AP15 was provided by Selleckchem (United States). LPS (from *Escherichia coli*, 055: B5), Phorbol 12-myristate 13-acetate (PMA), and Brewer thioglycollate medium were obtained from Sigma–Aldrich (United States). Alexa Fluor 647 phalloidin and primary antibodies were raised against IκBα, ERK1/2, JNK, p38 MAPK, NF-κB P65, PCNA, and GAPDH, and the phosphorylated ERK (Thr202/Tyr204), JNK (Thr183/Tyr185), and p38 MAPK (Thr180/Tyr182) were manufactured by Cell Signaling Technology (United States). The CellTiter 96 Aqueous One Solution reagent used in MTS assays was provided by Promega Corporation (United States). Nuclear and cytoplasmic protein extraction kits were obtained from Keygen Company (China). DAPI was purchased from Abcam (United Kingdom). Enhanced chemiluminescence (ECL) reagents were provided by Santa Cruz Biotechnology (United States). The tumor necrosis factor alpha (TNF-α) and interleukin 6 (IL-6) ELISA kits of mouse and human were obtained from Dakewe Biotechnology (China).

### Animal Model

All animals were raised and treated according to the ethical guidelines of animals of the Guangdong Animal Center. The protocols of animal experiments were approved by the Institutional Animal Care and Use Committee of Guangzhou Medical University (Guangzhou, China). All animals were kept in standard environment and were fed with standard laboratory diet and water. Six- to 8-week-old male C57BL/6 mice were obtained from the Guangdong Medical Laboratory Animal Center. The mice were divided randomly into four groups, with 15 in each group. The mice in the experimental group were administered with b-AP15 (5 mg/kg) (Cai et al., 2017) or empty vehicle (DMSO: Cremophor: 0.9% sodium chloride = 1:3:6) by intraperitoneal injection 2 h before they were administered intraperitoneally with LPS (25 mg/kg). The mice in the control group were given empty vehicle by intraperitoneal injection. In each group, three mice were anesthetized by isoflurane when they were given LPS treatment 6 h later. Then they were sacrificed for experimental samples by cervical vertebra dislocation. The remaining mice were used to evaluate the survival rate after LPS treatment. Mortality was evaluated 10 days after LPS treatment.

### Serum and Bronchoalveolar Lavage Fluid (BALF) Collection

Blood from the eyes was collected in mice under anesthesia. The blood was stored for 30 min at room temperature and then was centrifuged to collect the upper serum. After the mice were euthanized, the lungs were syringed with 500 μl of ice-cold PBS thrice through the tracheal cannula. The upper clear liquid was collected as BALF after the lavage fluid was centrifuged (Van Hoecke et al., 2017). Both serum and BALF were stored at −20°C until use.

### Cell Culture

The human monocytic leukemia THP-1 cells were provided by the American Type Culture Collection (ATCC, United States). The mouse macrophage cells were taken from the peritoneum of male C57BL/6 mouse, and the experimental protocols were performed as described previously (Zhang et al., 2008). Male C57BL/6 mice were intraperitoneally injected with 3% Brewer thioglycollate medium. The mice were euthanized after 3 days and intraperitoneally injected with 5 ml PBS (Gibco, United States) to collect peritoneal macrophages. Mouse peritoneal macrophages were maintained in DMEM (Gibco, United States), and THP-1 was maintained in RPMI 1640 medium (Gibco, United States). Both mediums contained 10% fetal bovine serum (FBS; Biological Industries, Israel), 2 mM L-glutamine, 100 μg/ml streptomycin, and 100 U/ml penicillin (Gibco, United States). The PMA (100 ng/ml) was used to induce THP-1 differentiation for 24 h before cell experiments. All cells were placed in an incubator (Thermo Fisher, United States) at 37°C in a humid environment and 5% CO_2_.

### Cell Viability Assessment

MTS assay was performed to evaluate cell viability according to the application manual. Firstly, sterile 96-well culture plates were chosen, and THP-1 and mouse peritoneal macrophages were seeded in it according to appropriate density. After the cells were treated, 20 μl MTS solution was added to each well. A microtiter plate reader was used to gain absorbance at a wavelength of 490 nm.

### Cytokine Level Determination

THP-1 and mouse peritoneal macrophages were seeded in different 24-well culture plates. After the cells were stimulated by LPS (100 ng/ml) and different concentrations (0.125, 0.25, 0.5, 0.75, or 1 μM) of b-AP15 for 6, 12, and 24 h, corresponding cell culture supernatants were collected by centrifugation. The mouse serum and BALF were collected as described above. The cytokine levels of cell supernatants, mouse serum, and BALF were assessed by ELISA according to the manufacturer’s instructions to quantify the amounts of IL-6 and TNF-α.

### Cytoplasmic and Nuclear Protein Extraction

To obtain proteins from cell cytoplasm and nucleus, THP-1 and mouse peritoneal macrophages were seeded in 6-cm cell culture plates. After the cells were treated with LPS (100 ng/ml) and different concentrations (0.5 or 1 μM) of b-AP15 for 30 min, the cytoplasmic and nuclear proteins were extracted according to the manufacturer’s protocol. The Nuclear and Cytoplasmic Protein Extraction Kit (Keygen, China) contains Buffer A, Buffer B, Buffer C, 100 mM PMSF, and 1000 × PI. First, the cells were washed by ice-cold PBS twice and collected by centrifuge (4°C, 800 rcf, 3 min). After supernatants were discarded, the cells were added with moderate Buffer A and Buffer B (Buffer A: Buffer B = 9:1, and per milliliter of Buffer A contains 17 μl 100 mM PMSF and 1 μl PI). Both of them were involved in incubation for 30 min on the ice after they were mixed. Next, supernatants were collected by centrifuge (4°C, 3000 r/min, 10 min) as extracts of cytoplasmic proteins. The sediments were mixed with moderate Buffer C (per milliliter of Buffer C contains 17 μl 100 mM PMSF and 1 μl PI) and were oscillated sharply for 15 s. The mixtures were involved in incubation for 30–60 min on the ice and were oscillated sharply for 15 s per 10 min. Finally, supernatants were collected by centrifuge (4°C, 14,000 rcf, 30 min) as extracts of nuclear proteins.

### Western Blot Analysis

In brief, cells were dissolved in cell lysis buffer to obtain total protein. The amounts of total protein were evaluated by bicinchoninic acid (BCA) assay (Thermo Fisher, United States). The cells were then placed on 12% SDS-PAGE to separate the total protein. Total protein solution was added to each well on SDS-PAGE equally. The total proteins were then electro-transferred onto methanol-treated polyvinylidene fluoride (PVDF) membranes (Merck Millipore, United States). Next, 5% non-fat milk was dissolved in PBS containing 0.1% Tween-20 (PBST), which was used for blocking the blots. The blots were then incubated with suitable primary antibodies at 4°C overnight after blocking. Next day, the secondary antibodies were added to the incubation box where the blots were present for 1 h at room temperature. The expression level of the target protein was determined by an ECL kit and autoradiography.

### Confocal Analysis

Following the previous steps (Liu et al., 2017), THP-1 and mouse peritoneal macrophages were seeded in 24-well culture plates with 14-mm circular coverslips. After treatment, the cells were fixed by 4% paraformaldehyde (PFA) and then were blocked with 5% BSA. After that, the cells were incubated against NF-κB P65 antibodies (1:200) at 4°C overnight. The following day, fluorescein-labeled goat anti-rabbit IgGs (Alexa Fluor 647 phalloidin; DyLight 647; 1:200) were added for 1 h in a dark place. The cells were then counterstained with DAPI. Observation and analyses were carried out by a confocal laser scanning microscope (SP8, Leica, Germany).

### Statistical Analyses

Experimental primary data were analyzed by professional SPSS 16.0 software. Data were presented as mean ± standard deviation (SD). The differences between the groups were evaluated by one-way analysis of variance (ANOVA) and *t*-test. A *P*-value of < 0.05 was considered to be statistically significant.

## Results

### b-AP15 Significantly Reduced the Levels of LPS-Stimulated Proinflammatory Cytokines in THP-1 and Mouse Peritoneal Macrophages

**FIGURE 1:**
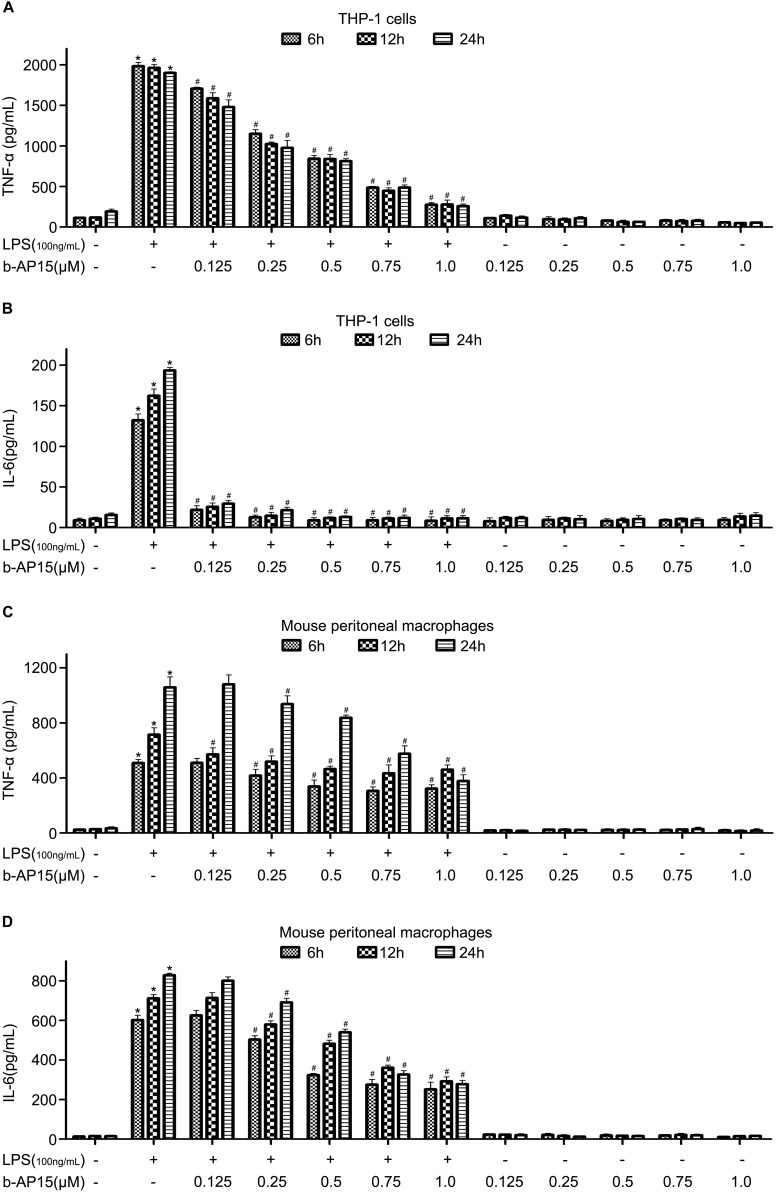
The effect of b-AP15 on the levels of LPS-stimulated proinflammatory cytokines in THP-1 and mouse peritoneal macrophages. **(A–D)** THP-1 and mouse peritoneal macrophages were incubated with DMSO, LPS (100 ng/ml), LPS + b-AP15 (0.125, 0.25, 0.5, 0.75, or 1.0 μmol/l), and b-AP15 (0.125, 0.25, 0.5, 0.75, or 1.0 μmol/l) for 6, 12, and 24 h. The production of IL-6 and TNF-α was determined by ELISA. All experiments were performed at least three times, and the data were presented as mean ± standard deviation (SD). *P < 0.05 compared with the vehicle control-treated group. ^#^*P* < 0.05 compared with the LPS-treated group.

TNF-α and IL-6 are the most common indexes for inflammation. To determine whether b-AP15 has an effect on TNF-α and IL-6 production, THP-1 and mouse peritoneal macrophage cells were chosen. The amounts of TNF-α and IL-6 were measured by ELISA assay after the cells were stimulated by LPS and treated by b-AP15. The results were presented in Figure 1, and the amounts of TNF-α and IL-6 were increased when THP-1 was stimulated by LPS (100 ng/ml) for 6, 12, and 24 h. However, both were significantly decreased after the cells were treated by b-AP15 (Figures 1A,B). The results obtained from mouse peritoneal macrophages showed similar results (Figures 1C,D). All these demonstrated that b-AP15 has a positive anti-inflammatory action *in vitro* and that it might act as a negative regulator of LPS-induced inflammation by inhibiting the DUB activity.

### b-AP15 Caused No Obvious Cell Death in THP-1 and Mouse Peritoneal Macrophage Cells in Low Concentrations

**FIGURE 2:**
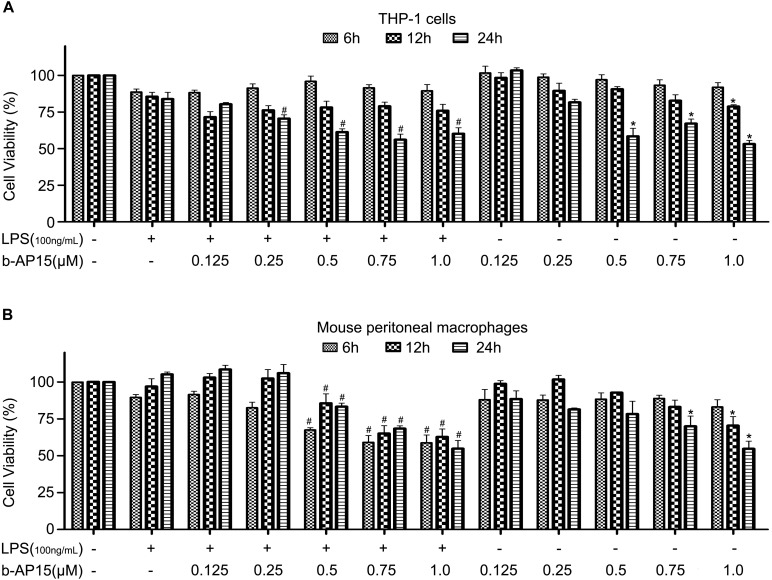
The effect of b-AP15 on cell viability of THP-1 and mouse peritoneal macrophages. **(A,B)** THP-1 and mouse peritoneal macrophages were incubated with DMSO, LPS (100 ng/ml), LPS + b-AP15 (0.125, 0.25, 0.5, 0.75, or 1.0 μmol/l), and b-AP15 (0.125, 0.25, 0.5, 0.75, or 1.0 μmol/l) for 6, 12, and 24 h. Cell viability was evaluated by MTS assay. The experiments were performed at least three times, and data were presented as mean ± standard deviation (SD). *P < 0.05 compared with the vehicle control-treated group. ^#^*P* < 0.05 compared with the LPS-treated group.

The cytotoxic effect of b-AP15 was assessed to exclude the decrease of proinflammatory cytokines by b-AP15 due to the cell death effect. The cell viability of THP-1 and mouse peritoneal macrophages was evaluated by MTS assay. The cells were treated with LPS (100 ng/ml) and different concentrations (0.125, 0.25, 0.5, 0.75, or 1 μM) of b-AP15 for 6, 12, and 24 h. The results (Figure 2) showed that b-AP15 has some cytotoxic effects in high concentrations, but it is not so bad, because previous studies have reported that macrophage apoptosis is associated with inflammation and the rise of macrophage apoptosis helps to increase anti-inflammation effects. Therefore, promoting death of proinflammatory cells is a widely accepted treatment strategy for anti-inflammation (Marriott et al., 2006; Lu et al., 2011).

### b-AP15 Attenuated LPS-Induced Inflammation by Suppressing ERK1/2 and JNK Phosphorylation in THP-1 and Mouse Peritoneal Macrophages

**FIGURE 3:**
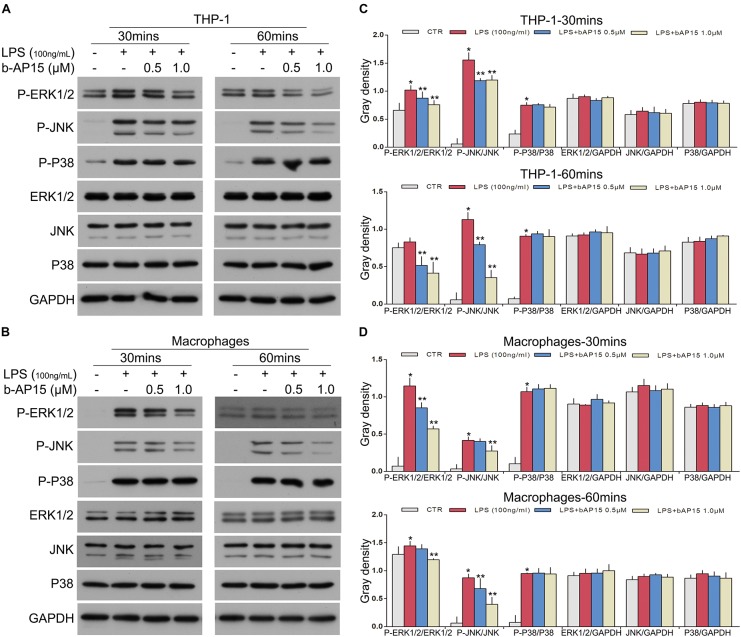
b-AP15 regulates the activation of THE MAPK signaling pathway in THP-1 and mouse peritoneal macrophages. **(A,B)** THP-1 and mouse peritoneal macrophages were incubated with DMSO, LPS (100 ng/ml), and LPS + b-AP15 (0.5 or 1.0 μmol/l) for 30 and 60 min. The total proteins in cells were extracted by cell lysis buffer. The P-ERK1/2, P-JNK, P-P38, ERK1/2, JNK, and P38 levels in cell lysates were detected by immunoblotting, and GAPDH was used as a normalization factor. **(C,D)** Quantification of band gray density was counted. *P < 0.05 vs. the vehicle control-treated group, **P < 0.05 vs. the LPS-treated group. Three independent experiments were performed.

Inflammatory reaction was regulated by multiple signal transduction pathways, and the MAPK signaling pathway plays a crucial role in inflammatory reactions (Arthur and Ley, 2013; Paunovic and Harnett, 2013). Therefore, we hypothesized whether b-AP15 treatment impacts the activation of MAPK. Western blotting was performed to evaluate ERK1/2, JNK, and P38 activation (phosphorylation) in cells. The results revealed that b-AP15 significantly reduced ERK1/2 and JNK phosphorylation in LPS-stimulated THP-1. However, no influence was observed upon p38 MAPK phosphorylation (Figures 3A,C). Meanwhile, mouse peritoneal macrophages were also used to verify our hypothesis, and similar results were obtained (Figures 3B,D). These results indicated that b-AP15 reduced LPS-induced inflammation via suppression of ERK1/2 and JNK phosphorylation in THP-1 and mouse peritoneal macrophages.

### b-AP15 Reduced LPS-Induced Inflammation by Increasing IκBα Activation and Inhibiting NF-κB Activation in THP-1 and Mouse Peritoneal Macrophage Cells

**FIGURE 4:**
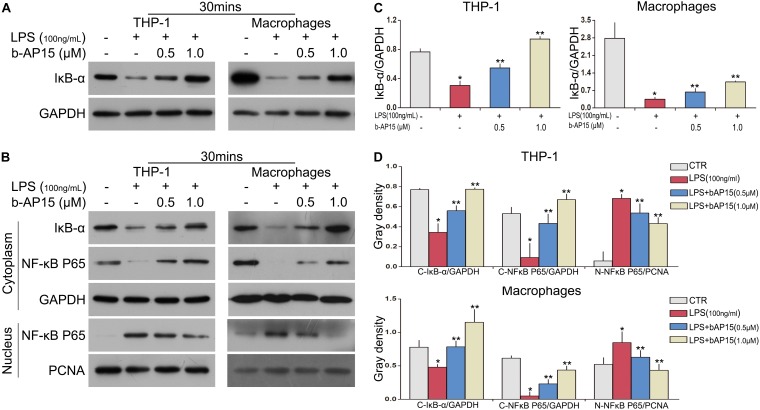
b-AP15 regulates the activation of the NF-κB pathway in THP-1 and mouse peritoneal macrophages. THP-1 and mouse peritoneal macrophages were incubated with DMSO, LPS (100 ng/ml), and LPS + b-AP15 (0.5 or 1.0 μmol/l) for 30 min. **(A)** The total proteins in cells were extracted by cell lysis buffer. The level of IκB-α in cell lysates was detected by immunoblotting, and GAPDH was used as a normalization factor. **(B)** The protein lysates of the cytoplasm and nucleus were used for western blotting of NF-κB p65 and IκBα proteins. GAPDH and PCNA were selected as normalization factors. **(C,D)** Quantification of band gray density were counted. *P < 0.05 vs. the vehicle control-treated group, **P < 0.05 vs. the LPS-treated group. Three independent experiments were performed.

**FIGURE 5:**
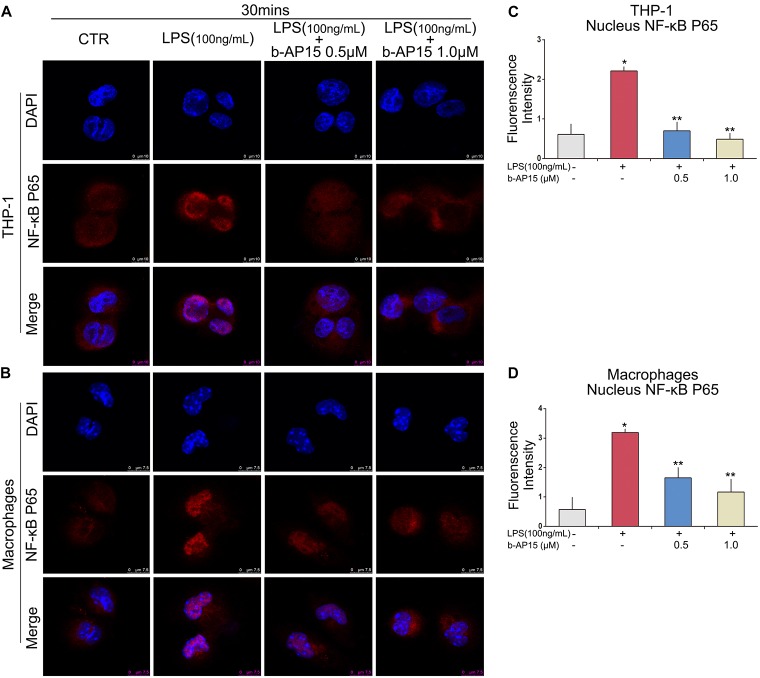
Effects of b-AP15 on LPS-dependent NF-κB pathway activation in cells. THP-1 **(A)** and mouse peritoneal macrophages **(B)** were incubated with DMSO, LPS (100 ng/ml), and LPS + b-AP15 (0.5 or 1.0 μmol/L) for 30 min. Immunofluorescence and confocal microscopy were performed to assess NF-κB p65 translocation. **(C,D)** Quantification of fluorescence intensity was counted by ImageJ. *P < 0.05 vs. the vehicle control-treated group, **P < 0.05 vs. the LPS-treated group. Three independent experiments were performed.

The NF-κB signaling pathway is an important signaling pathway in LPS-induced inflammatory reactions (Gilmore, 2006; Carmody and Chen, 2007; Hayden and Ghosh, 2012). Besides the MAPK signaling pathway, it is necessary to explore whether b-AP15 regulates the activation of NF-κB in THP-1 and mouse peritoneal macrophages. Western blotting was performed, and the results showed that the LPS-treated cells demonstrated decreased expression of IκBα. However, after b-AP15 treatment, the LPS-mediated inflammatory reactions were suppressed by increased expression of IκBα (Figures 4A,C). Next, the protein in the nucleus and cytoplasm was also analyzed, and the results revealed that LPS-treated cells had enhanced process of NF-κB P65 translocation from the cytoplasm to the nucleus, while the nuclear translocation of NF-κB p65 was reduced after b-AP15 treatment (Figures 4B,D). In addition, similar results were observed by confocal microscopy, which clearly shows that the nuclear translocation phenomenon of NF-κB p65 in THP-1 and mouse peritoneal macrophages was decreased by b-AP15 treatment (Figure 5). These results suggested that b-AP15 negatively regulates LPS-induced inflammatory response by inhibiting the activation of the NF-κB signaling pathway.

### Anti-inflammatory Role of b-AP15 *in vivo*

**FIGURE 6:**
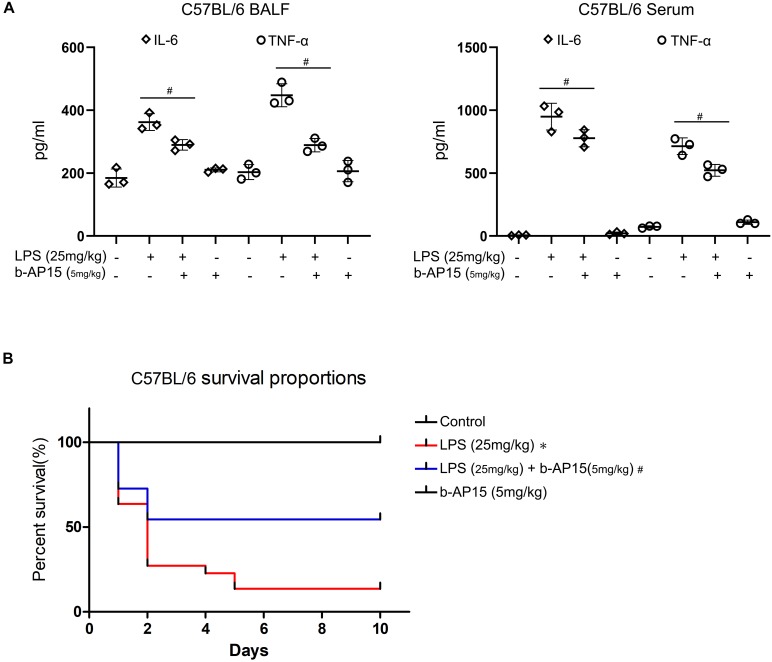
b-AP15 anti-inflammatory action *in vivo*. **(A)** C57BL/6 mice were incubated with b-AP15 (5 mg/kg) for 2 h before LPS administration (25 mg/kg) for 6 h. The BALF and serum were collected to test the amounts of IL-6 and TNF-α by ELISA. Data were presented as mean ± standard deviation (SD). *P < 0.05 compared with the vehicle control-treated group. ^#^*P* < 0.05 compared with the LPS-treated group. **(B)** After the mice were treated with b-AP15 (5 mg/kg) for 2 h, they were administered with LPS (25 mg/kg) for one time. During a 10-day observation period, the number of dead mice was collected to evaluate the survival rate. The differences between the groups were examined by one-way ANOVA. *P < 0.05 compared with the vehicle control-treated group. ^#^*P* < 0.05 compared with the LPS-treated group. Three independent experiments were performed.

To further verify the anti-inflammatory effect of b-AP15, related experiments were conducted *in vivo*. Male C57BL/6 mice were used to build a sepsis model by treatment with high-density LPS (25 mg/kg). ELISA was performed to assess the levels of proinflammatory cytokines in the mice. The results showed high levels of proinflammatory cytokines TNF-α and IL-6 in BALF of sepsis mice without b-AP15 treatment when compared with controls. However, the mice pretreated with b-AP15 showed lower levels of TNF-α and IL-6 (Figure 6A). Also, the serum of mice was analyzed, and the results are the same as those obtained in BALF (Figure 6A). In addition, survival rate analysis was performed in the sepsis mice. During a 10-day observation period, there were no deaths observed in the groups that were given empty vehicle or b-AP15 treatment alone, showing a 100% survival rate. However, the survival rate of the group that was given LPS alone was only 13.6%, and the group administered with LPS and b-AP15 showed a higher survival rate of 54.5% (Figure 6B). This clearly indicated that b-AP15 not only has a great anti-inflammatory effect *in vitro* but also has a good anti-inflammatory action *in vivo* for mice with LPS-induced inflammation. Both are considered as an important reference in clinically guiding microbial infection.

## Discussion

Inflammation is a common symptom observed in patients clinically. Besides microbial infection, some acute injuries, autoimmune diseases, and chronic diseases also lead to inflammatory responses. Therefore, inflammation is worthy to be discussed and is also an urgent problem to be resolved (Brouwers et al., 2015; Prabhu and Frangogiannis, 2016; Robb et al., 2016). We herein described the complex inflammatory mechanism, and activation of macrophages is considered critical for inflammatory responses. Generally, it is necessary to control the production of proinflammatory cytokines to reduce inflammation responses by inhibiting the activation of macrophages. And there are so many chemical means of anti-inflammation, such as exosomes, natural extracts, and so on (Ti et al., 2015; Chen et al., 2019). But the regulatory effect of the DUB inhibitor in inflammatory signal transduction pathways still remains unclear. Nowadays, several DUB inhibitors showed enormous potential as targeted drugs in clinics. For example, bortezomib has been used as a drug target successfully in clinical practice (D’Arcy and Linder, 2012). Similarly, b-AP15 as a DUB inhibitor also has huge potential as an anti-inflammatory agent. According to a study, b-AP15 promoted cell apoptosis of cancer cells by DR5 activation and enhanced the cancer sensibility for anticancer treatment (Oh et al., 2017). b-AP15 overcomes drug resistance of multiple myeloma for bortezomib (Tian et al., 2014). Meanwhile, it also promoted cell death of multiple myeloma cells via the caspase-dependent apoptotic signaling pathway, and other researchers obtained similar results in human mantle cell lymphoma (MCL) (Kropp et al., 2018). In liver cancer, b-AP15 kills human liver cells by enhancing ER stress and blocking the Wnt/Notch1 pathway (Ding et al., 2018). Besides, a significant anticancer effect has been observed when breast cancer cells with ERα overexpression were treated by PtPT and b-AP15 (Xia et al., 2018). Therefore, b-AP15 has exhibited excellent prospects obviously. According to our results above, the DUB inhibitor b-AP15 inhibited the activation of macrophages successfully, reducing the levels of proinflammatory cytokines TNF-α and IL-6 in both *in vitro* and *in vivo* experiments.

Poltorak et al. (1998) found that Toll-like receptor 4 (TLR4) is a receptor for LPS in mammals. LPS binds to TLR4, which induces the activation of intracellular signal transduction networks quickly, including the MAPK pathway and NF-κB pathway. These signaling pathways activate a variety of transcription factors, which promote proinflammatory cytokine expression (Guha and Mackman, 2001). In mammals, the majority of MAPK pathways, along with the NF-κB pathway, were recruited by stress and inflammatory stimuli. Accordingly, these pathways represent a substantial trove of potentially important targets for anti-inflammatory material (Kyriakis and Avruch, 2012). These showed the important roles of MAPK and NF-κB in anti-inflammation research. In fact, many studies have reported that the inhibition of MAPK or NF-kB activation suppressed LPS-induced inflammation in macrophages (Dong et al., 2019; Kim et al., 2019; Ma et al., 2019; Shin et al., 2019; Somensi et al., 2019; Tian et al., 2019). Similarly, here, we found that b-AP15 inhibited phosphorylation of ERK1/2 and JNK and suppressed inflammatory reactions in THP-1 and mouse peritoneal macrophages. The ERK1/2, JNK, and p38 MAPK signaling molecules belong to the MAPK pathway, and both of them were phosphorylated rapidly in LPS-stimulated macrophages. The MAPK pathway has been reported as a classical signaling pathway of inflammation by some scholars (Arthur and Ley, 2013; Paunovic and Harnett, 2013). Obviously, b-AP15 inhibits ERK1/2 and JNK phosphorylation and suppresses the inflammatory reactions, which are processes that are not mutually isolated but have a close relationship between them. Unfortunately, we did not further explore how b-AP15 works on ERK1/2 and JNK. However, our discovery provides a new perspective on how a DUB inhibitor regulates the signal transduction of the MAPK pathway in inflammation.

In addition, interestingly, b-AP15 significantly increased the levels of IκBα and blocked the activation of NF-κB in THP-1 and mouse peritoneal macrophage cells. Normally, DUB inhibitors play a promotive role in protein degradation. Here, in contrast, b-AP15 increased IκBα expression. Mialki et al. found that USP14 reduced the levels of polyubiquitinated IκB, which promoted the deubiquitinated IκB degradation within the proteasome. Meanwhile, USP14 increased the LPS-induced cytokine release in lung epithelial cells (Mialki et al., 2013). Besides, Li et al. (2019) also found that USP14 regulates the IκBα by promoting its deubiquitination and degradation in chondrocytes. And there is an interaction between these two proteins. In addition, LPS treatment induced serine phosphorylation of USP14 quickly (Mialki et al., 2013). A previous study has reported that serine phosphorylation of USP14 showed high DUB activity (Xu et al., 2015). USP14 as a regulatory DUB of 19S RP plays important catalytic and allosteric roles in proteasomal degradation (Chadchankar et al., 2019). It activates the proteasome through a DUB activity-independent manner (Peth et al., 2009). Therefore, the proteasome function could be injured when USP14 has been targeted by b-AP15. In fact, b-AP15 inhibits the proteasome function, as reported in many studies of cancer. D’Arcy and Linder (2012) and Brnjic et al. (2014) pointed out that exposure to b-AP15 results in blocking of the proteasome function, which results in accumulation of polyubiquitin in some cancer cells. Some ubiquitin-mediated protein degradation was reversed by b-AP15 in TNBC (Vogel et al., 2015). Meanwhile, a previous study has shown that exposure to b-AP15 results in the accumulation of Ub and K48 quickly. This indicates that b-AP15 inhibits the function of proteasome very quickly (Jiang et al., 2019). Similarly, the DUB inhibitor Auranofin can inhibit USP14 and ubiquitin C-terminal hydrolase 5 (UCHL5) as b-AP15 does. We have reported that Auranofin treatment increased the accumulation of Ub, K48, and K63 and suppressed the degradation of IκBα too. Auranofin blocks ubiquitin-mediated protein degradation through suppressing the proteasome function too (Liu et al., 2014; Hu et al., 2018). So we guess that probably b-AP15 stabilizes IκBα expression by suppressing proteasomal degradation, which inhibits the activation of NF-κB. As we all know, the NF-κB pathway also plays a vital role in inflammatory response, which is probably considered as a drug target for treating inflammation (Gilmore, 2006; Carmody and Chen, 2007; Hayden and Ghosh, 2012). These indicated that b-AP15 regulates proinflammatory cytokines IL-6 and TNFα by regulating the NF-κB signal transduction pathway, and this also plays a key role in the anti-inflammatory mechanism. Furthermore, the enormous anti-inflammatory value of b-AP15 was confirmed by the *in vivo* experiments. This might be of great significance for clinical guidance in the future.

In a word, our work proved that the DUB inhibitor b-AP15 reduced the level of proinflammatory cytokines TNF-α and IL-6 via ERK1/2 and JNK phosphorylation and inhibited NF-κB activation in LPS-induced THP-1 and mouse peritoneal macrophage. Therefore, b-AP15 is considered to be a potential anti-inflammatory drug in microbial infection. However, the inflammatory response is an extremely complex process with the involvement of multiple signaling pathways. The comprehensive molecular mechanism processes of b-AP15 anti-inflammatory action in cells and animals should be analyzed.

## Data Availability Statement

All the data and materials supporting the conclusions were included in the main manuscript.

## Ethics Statement

The use and care of experimental animals were approved by the Institutional Animal Care and Use Committee of Guangzhou Medical University.

## Author Contributions

NL and ZZ designed the experiments and wrote the manuscript. FZ, RX, RC, QX, ML, XkC, XhC, TK, CZ, and S-ML performed the experiments. All authors read and approved the final manuscript.

## Conflict of Interest

The authors declare that the research was conducted in the absence of any commercial or financial relationships that could be construed as a potential conflict of interest.
